# Queries Proposed to Those Medical Gentlemen Who Have Opportunities of Observing the Epidemical Ophthalmia Which Has Long Prevailed in the British Army

**Published:** 1810-10

**Authors:** Gegrge Joseph Beer

**Affiliations:** Imperial Oculist to the University and Institutions for the Relief of the Poor of the City of Vienna, Member of the Royal Society of Sciences of Gottingen, and Honorary Member of the Bohemian Humane Society of Prague


*Queries proposed to those medical gentlemen who have opportunities of observing the Epidemical Ophthalmia which has long prevailed in the British army. By* GEGRGE JOSEPH BEER, M. D. *Imperial Oculist to the University and Institutions for the Relief of the Poor of the City of Vienna, Member of the Royal Society of Sciences of Gottingen, and Honorary Member of the Bohemian Humane Society of Prague.*

THE various reports which begin to circulate upon the continent relative to an epidemical ophthalmia which has committed such dreadful ravages among the British soldiers, cannot but make a deep impression upon a person who, from his vocation as physician exclusively employed in the treatment of the diseases of the eyes in a populous town like Vienna, is peculiarly situated to appreciate the advantages of sight, and who is daily under the necessity of undertaking the painful task of declaring a number of unfortunate victims blind without possibility of relief. This consideration alone will justify me in venturing to propose some important questions to the British tish physicians who are intrusted by their government with the treatment of this epidemical disease. These questions will enable me, perhaps, though at a great distance from the places where that dreadful evil rages, to make some useful proposals which may tend more quickly to moderate its violence, and finally to eradicate it.

How happy should I think myself if I could in this manner not only contribute to save the eyes of a part of the British army, but even, perhaps, acquit a share of the great debt which Germany has contracted with England for the incalculable benefits of vaccination!

The questions upon the exact answers of which depends the possibility of acquiring a precise knowledge of the epidemical ophthalmia which rages among British soldiers are the following:

1. How long is it since this epidemy has first made its appearance?

2. In what country was it first observed?

3. Has any peculiar cause been observed to which this disease may be attributed, either in those places where it first made its appearance, or in those where it propagated itself afterwards?

N. B. In answering this query, particular attention must be made to climate, to situation, to the changes of temperature, to the nature of the dust which is thrown in the eyes, to the water which is used daily for washing the eyes, to the articles of food, to the various dresses which are in contact with the head, for instance, neck-cloths, head ornaments, hats, caps, and the like, and still more particularly, to the manner of living of the inhabitants, and lastly, to the insects found in those places.

4. Does this epidemy remain fixed in one quarter? or does it spread gradually in the surrounding places? and if so, in which direction?

5. Is the propagation of the epidemy (if it really take place) a consequence of certain changes of the atmosphere, for instance, of damp weather? of violent winds?

6. Has the epidemical ophthalmia raged hitherto always with equal violence? or has it shewn sometimes evident remissions?

7. If those remissions have really been observed, in what weather have they occurred? and has the same state of the weather always been found to produce the same remissions?

8. Is it likely, or has it been ascertained that this ophthalmia is contagious? and what are the circumstances which facilitate and accelerate the contagion?

9. Has

9. Has there been hitherto any remedy for this disease, praised by the vulgar, as a preservative, or even acknowledged as such by physicians, with some degree of foundation? and what is it?

10. Is the course of this epidemical ophthalmia quick, or slow?

11. Does any difference in the rapidity of its course manifest itself sometimes in various individuals?

12. Has it been observed that this disease attacks principally a certain class of constitutions (organisms) or only particular habits, whilst it spares others? or does it attack every individual who comes near enough to the sphere of its action, without difference of temperament, of age, of profession, and of rank?

13. As it is however impossible that every individual constitution could be attacked with equal force by this epidemical disease of the eyes, because every external effect must be relative, it is asked; which constitution suffer most violently, and in which it is most dangerous?

14. Is this ophthalmia always, or only in some subjects attended with a general disease of the whole frame?

15. Does that constitutional disease (if it exist) manifest itself before the ophthalmia, or only during its course? and in which period of it?

16. What is the intensity and the duration of that constitutional disease?

17. What are its principal symptoms and periods?

18. Is it attended with considerable febrile symptoms?

19. Is the fever under the influence of which the constitutional disease takes place, continued, remittent, or intermittent?

20. If a constitutional disease accompany this ophthalmia only in some individuals, what is in general their habit?

21. What is the succession of the morbid symptoms which are observed from the beginning to the end of the ophthalmia?

N. B. The undersigned begs particularly the medical gentlemen to whom this question is proposed, to pay the greatest attention to the species, the duration, the intensity and the seat of the pain; to the various degrees of derangement of the functions of the eye, viz. to the state of the power of sight during the ophthalmia? to the various developments of light before the eye; to the aversion to light, &c. in one word, to all morbid phenomena in the eyes and surrounding parts, which collectively have some influence upon the sensations of the patient?

22. What is the succession of the morbid phenomena which takes place from the beginning to the end of this ophthalmia in the organic substance and form of the eye?

N. B. To

N. B. To render the answer to this question perfectly satisfactory to the consulting oculist, it is necessary to describe with the utmost care which parts of the eye are attacked at the beginning of the ophthalmia, and to know whether the inflammatory action begins in the noble and most subtile parts of the eye, viz. in the retina, choroides, iris, and so forth, and proceeds from the internal to the external parts which surround the eye; or whether at first the external part of the eye, viz. the eye lids, the conjunctiva, the sclerotica, and the cornea are attacked by the ophthalmia; and whether consequently the direction of the inflammatory action propagates itself gradually from outwards to the inside of the eye?

2° In answering this question, it will be particularly necessary to examine, whether in the course of the ophthalmia a true suppuration occur, and in which part of the eye it takes place, or whether this ophthalmia follows the course of a merely adhesive inflammation; or lastly, whether visible extravasations of lymph or pus take place in the chambers of the eye, which gradually acquire an organisation, and produce unnatural adhesions of the different parts of the eye, for instance, of the uvea with the capsule of the crystalline lens, of the iris with the cornea, etc.

23. Is blindness the invariable consequence of this disease? or is there any example of patients undergoing it without loosing the power of sight?

24. If this last sometimes happen, we ask, under which treatment is the eye most easily saved, and under which treatment is the sight most often, most easily, and most quickly lost?

25. Is the sight lost by a complete destruction of the globe of the eye *(colliquatio, disorganisatio)* or by the disorganisation of some parts of the eye? and which are those parts?

26. In the last case, viz. when the sight is lost by the destruction of individual parts of the eye, is the blind subject still capable of some perception of light? and in what degree?

27. With those individuals which are happily cured, that is to say, those who still enjoy the faculty of seeing, does any defect in the appearance and the form of the eye remain?

28. Is the recovery usually slow, and with what symptoms is it attended?

29. Does the recovery require any particular treatment in order to avoid a relapse?

*Vienna, November* 6, 1806.

